# Microbiological Screening Is Necessary to Distinguish Carriers of Plasmid-Mediated AmpC Beta-Lactamase-Producing Enterobacteriaceae and Extended-Spectrum Beta-Lactamase (ESBL)-Producing Enterobacteriaceae because of Clinical Similarity

**DOI:** 10.1371/journal.pone.0120688

**Published:** 2015-03-24

**Authors:** Anna Conen, Reno Frei, Hildegard Adler, Marc Dangel, Christoph A. Fux, Andreas F. Widmer

**Affiliations:** 1 Division of Infectious Diseases and Hospital Epidemiology, Kantonsspital Aarau, Aarau, Switzerland; 2 Division of Clinical Microbiology, University Hospital Basel, Basel, Switzerland; 3 Division of Infectious Diseases and Hospital Epidemiology, University Hospital Basel, Basel, Switzerland; Amphia Ziekenhuis, NETHERLANDS

## Abstract

**Objectives:**

Plasmid-mediated AmpC beta-lactamase-producing (pAmpC) Enterobacteriaceae are increasing worldwide, difficult to identify and often confounded with extended-spectrum beta-lactamase (ESBL) producers. The low prevalence precludes routine universal admission screening. Therefore, we evaluated potential risk factors for carriage of pAmpC-producing Enterobacteriaceae that would allow targeted screening to improve yield and reduce cost.

**Patients and methods:**

We performed a case control study at a tertiary care center from 1/2006 to 12/2010. Cases were adult patients in whom pAmpC-producing Enterobacteriaceae were isolated; controls were chosen among carriers of ESBL-producing Enterobacteriaceae. Both infected and colonized patients were included.

**Results:**

Over five years, we identified 40 pAmpC producers in 39 patients among 16,247 screened consecutive isolates of Enterobacteriaceae. The pAmpC prevalence was low (0.25%), but more than 30% of pAmpC carriers received incorrect empirical antibiotic treatment. When compared with 39 ESBL controls, pAmpC carriage was associated with clinically confirmed infections in 74% (versus 51%) (p=0.035), mainly of the urinary tract, previous antibiotic exposure in 63% (versus 36%) (p=0.035) and carriage of a nasogastric tube in 23% (versus 0%) (p=0.002). In the multivariate regression analysis only clinically confirmed infections remained significantly associated with pAmpC carriage (OR 1.44 (95%CI 1.15-2.57)). No other clinical and blood test-associated risk factor allowed discrimination of pAmpC-carrying patients from ESBL controls. The type of acquisition – nosocomial versus community-acquired – was also non-informative for resistance type, as 46% of pAmpC- and 44% of ESBL-producing Enterobacteriaceae were community-acquired.

**Conclusions:**

This study could not identify a clinical profile that would allow targeted screening for pAmpC-producing Enterobacteriaceae when compared to ESBL carriers. Because empiric antimicrobial therapy was inappropriate in more than 30%, rapid identification of pAmpC carriers is needed. New microbiological methods are therefore required to simplify rapid and reliable detection of pAmpC carriers.

## Introduction

Multidrug-resistant Enterobacteriaceae (MRE) are rapidly emerging worldwide and account for a large proportion of health care-associated infections [1]. MRE include gram-negative bacteria expressing extended-spectrum beta-lactamases (ESBL), carbapenemases such as the *Klebsiella pneumoniae* carbapenemase (KPC), metallo-beta-lactamases such as the New Delhi metallo-beta-lactamase (NDM), and in addition, Enterobacteriaceae in which chromosomal AmpC beta-lactamases (cAmpC) can be induced by the exposition to beta-lactam antibiotics or mutants of Enterobacteriaceae in which AmpC-production is stably derepressed [2,3]. Rarely and less well documented is resistance to broad spectrum cephalosporins by plasmid-mediated AmpC (pAmpC).

pAmpC producers were first described in 1989. They have descended from c*ampC* genes and fall into six phylogenetic groups. Origins are the *ampC* genes of *Hafnia alvei*, *Morganella morganii*, *Citrobacter freundii*, *Enterobacter cloacae* and two as yet unidentified organisms. pAmpC are known to exist in various species lacking inducible c*ampC* genes including *Klebsiella* spp., *Proteus mirabilis*, *Salmonella enterica* and *Shigella* spp. *Escherichia coli* routinely carries c*ampC* gene*s*, which are very rarely hyperexpressed, and therefore, *E*.*coli* commonly responds to second and third generation cephalosporins. However, some strains negative for *campC* or those that almost never express cAmpC, may acquire p*ampC* genes rendering them resistant to these cephalosporins [4]. pAmpC producers are difficult to identify by means of the routinely performed microbiological analyses and may be misclassified as ESBL producers and vice versa [5,6]. Therefore, underreporting is likely [4,5,7]. Furthermore, pAmpC detection in the microbiology laboratory is time-consuming and costly [8]. In a recent publication from Germany, more than 50% of healthy broiler chicken were carriers of pAmpC-producing Enterobacteriaceae, potentially serving as reservoir for spread [9]. In humans, the prevalence of pAmpC-producing Enterobacteriaceae varies widely and is approximately 1%- 8.5% in the United States [10,11], 2%- 10% in Asian countries [12,13], 2.6% in the Netherlands [14], and 0.25% in the northern part of Switzerland [8]. Interestingly, a recent Swiss study demonstrated a particularly high prevalence of pAmpC in patients from specialized outpatient clinics (12.5%) [15].

The most prevalent pAmpC beta-lactamase is of CMY-2 type [14,16,17,18]. The majority of pAmpC producers is not susceptible to multiple antimicrobial substances including beta-lactamase inhibitors such as clavulanic acid, in contrary to ESBL producers. The susceptibilities for cefepime and carbapenems are usually not affected by pAmpC beta-lactamases [4,16].

Infections caused by ESBL producers have been associated with prolonged hospital stay, reduced rates of clinical and microbiological response to antimicrobial treatment and increased mortality [19,20]. For pAmpC-related infections, data are lacking or include only small patient numbers. Most patients with pAmpC producers suffer from co-morbidities (diabetes mellitus, chronic renal failure, abdomino-biliary diseases and neoplasia) and have undergone invasive procedures, such as insertion of urinary catheters (UC) or nasogastric tubes (NGT) or are mechanically ventilated, but both nosocomial- and community-related transmissions have been documented. Plasmid-mediated beta-lactamases might spread by a horizontal gene transfer [17,18,21,22,23,24,25].

A clinical profile that identifies patients at risk for pAmpC carriage would allow to select patients and to justify an in-depth microbiological analysis to detect pAmpC [12,17,18,22,23,26,27]. The low prevalence precludes routine universal admission screening, which is time-consuming and costly. The goal of the present study was therefore to characterize patients carrying pAmpC producers by clinical and laboratory parameters and to compare them with ESBL carriers as controls.

## Patients and Methods

We performed a case control study at a tertiary care centre in Basel, Switzerland. Cases were identified by analysing all consecutive clinical Enterobacteriaceae isolates without cAmpC for pAmpC production from 1/2006 to 12/2010.

Cases were adult patients older than 18 years in whom pAmpC-producing Enterobacteriaceae were isolated either as colonizing or infecting microorganisms. pAmpC carriers were identified from a microbiological database in which all PCR-positive pAmpC producers were collected [7]. They were excluded if no clinical data were available. Control patients were retrieved from the prospective surveillance of the Division of Hospital Epidemiology where all ESBL carriers during the study period (between 2006 and 2010) were routinely recorded, irrespective of whether the patients were infected or colonized [28]. They were selected by a computer-generated random table, matched in a 1:1 ratio according to age (+/-5 years), bacterial species and treating medical department. Clinical data were collected from patient charts by a board certified infectious diseases specialist using a case report form (CRF). The project was approved by the local ethical committee (Approval #112/12) (Ethikkommission beider Basel EKBB) who waived the necessity for individual informed consent.

### Case report form

The CRF included the following data: *socio-demographics* (age, gender, nationality, stay before admission: at home, nursing home, another hospital) and *presence of concurrent medical conditions* including chronic renal failure (estimated glomerular filtration rate <60 ml/min.) and haemodialysis, cardiovascular and chronic pulmonary disease, immunosuppression (defined as prednisone equivalent >10 mg daily for >3 months or similar), neoplasia, abdominal disease and diabetes mellitus. McCabe Score was calculated at hospital entry [29]. Data on hospitalizations and antibiotic exposure *within the 30 days prior* the date when the MRE was isolated were recorded. Data for the *time of detection of the MRE* included in- or outpatient treatment, and medical department where the patient was being treated: surgery, internal medicine, gynaecology and obstetrics, intensive care unit (ICU), the reason and duration of hospitalization, whether administered antibiotic treatment for the MRE was correct, and the use of indwelling medical devices at the time of the MRE isolation (UC, central venous catheter, NGT and mechanical ventilation). *Laboratory parameters* included inflammation markers (blood leukocyte count (normal if between 4,000 and 10,000 leukocytes/μl blood) and C-reactive protein (CRP, normal if <10 mg/l)) and liver function (elevated if >2 times upper normal limit; normal limit was for both GOT and GPT a level <40 U/l). *Microbiological data* included date and sources of samples that were positive for MRE: urinary tract, blood, abdominal-rectal, bone, respiratory secretions (exclusive detection in blood was defined as primary sepsis), species identification, and determination of the specific beta-lactamase by PCR. Antimicrobial susceptibilities to amoxicillin/clavulanic acid, piperacillin/tazobactam, cephalosporins, carbapenems (ertapenem was not included because it was not readily available during the study period), gentamicin, co-trimoxazole, nitrofurantoin, fluoroquinolones, and fosfomycin were recorded. Data on the empirical (prior the availability of the susceptibility testing) and targeted antibiotic *treatment* were collected and appropriateness of antimicrobial therapy was judged based on the susceptibility pattern.

### Definitions

Infection was identified according to the CDC classification for nosocomial infections [30]. Colonization with MRE was assumed if criteria for infection were not met. Acquisition was considered to be nosocomial, if the positive sample was obtained >48 hours after hospitalization [30]; and to be healthcare-associated if the MRE diagnosis was at the time of hospital admission or within 48 hours of admission and the patient fulfilled any of the following criteria: received intravenous therapy at home, wound or specialized nursing care through a health care agency or family, self-administered intravenous medical therapy, attended a hospital or haemodialysis clinic or received intravenous chemotherapy in the 30 days before the infection manifestation; was hospitalized in an acute care hospital for two or more days in the 90 days before the infection manifestation; or resided in a nursing home or long-term care facility [31]. Otherwise acquisition was community-acquired. Clinical cure was defined as the absence of infection at the time of hospital discharge. In-hospital mortality was related to infection if at the time of death signs of infection were present.

### Microbiology

Clinical isolates of Enterobacteriaceae of various species, which were lacking inducible c*ampC* genes, i.e. *E*. *coli*, *Klebsiella* spp., *P*. *mirabilis*, *S*. *enterica* ssp. *enterica* and *Shigella* spp., were screened for production of ESBL and plasmid-encoded AmpC. During the study period, the microbiology laboratory followed recommendations of the Clinical and Laboratory Standards Institute (CLSI) (formerly National Committee for Clinical Laboratory Standards) which remained unchanged [32]. All procedures in the microbiology laboratory remained unchanged as well.

For the microbiological detection of ESBL, standard culture methods were performed in accordance with the guidelines of CLSI [32]. Routine susceptibility testing was done using VITEK 2 (bioMérieux, Durham, NC) with the following compounds for ESBL screening: cefpodoxime, ceftriaxone, ceftazidime and aztreonam. If the screening test yielded a positive result, confirmation testing was performed with three different ESBL Etest strips containing cefotaxime, ceftazidime or cefepime with and without clavulanic acid (bioMérieux, Marcy l’Etoile, France, formerly AB Biodisk, Solna, Sweden). If the test result was positive, the type of the beta-lactamase was determined by PCR, as described earlier [28]. During the study period CLSI recommended to report ESBL always as non-susceptible to cephalosporins independent of the test result.

For the identification of pAmpC, the following procedure was used: Isolates that were positive in the ESBL screening test according to the CLSI guidelines were additionally tested for susceptibility to cefoxitin [32]. Isolates that were non-susceptible to cefoxitin were classified as putative pAmpC producers and further analysed by an AmpC multiplex PCR with primers specific for the genes of six different phylogenetic groups according to Pérez-Pérez and Hanson [7]. The amplicons were sequenced by use of a 3130 Genetic Analyser (Applied Biosystems, Foster City, Ca). Only isolates that tested positive by multiplex PCR according to Pérez-Pérez et al. were included as pAmpC producers [7].

### Statistical analyses

All analyses were done using SPSS (version 21.0). Data were entered into a spread sheet program (Excel; Microsoft) and then imported into SPSS. Cases and controls were compared by applying Mc Nemar tests. P-values less than 0.05 (two-tailed) were considered to be statistically significant. Uni- and multivariate logistic regression analyses were performed. Variables with p<0.1 were entered in the multivariate logistic regression model to adjust for multiple predictors. Results were presented as odds ratios (OR) and 95% confidence intervals (CI).

## Results

In the 5-year study period, 40 of 16,247 (0.25%) consecutive isolates of Enterobacteriaceae were positive in the pAmpC screening. The prevalence increased from 0.17% in 2006 to 0.31% in 2010 (table 1). None of the 39 patients was excluded. In one patient, two different pAmpC producers were isolated (*E*. *coli* and *K*. *pneumoniae*). *E*. *coli* with CMY-2 was the most common pAmpC producer. In 39 controls the most common ESBL producer was *E*. *coli* with CTX-M (table 2).

**Table 1:** Prevalence of plasmid-mediated AmpC beta-lactamase-producing Enterobacteriaceae from 2006 to 2010.

Year	2006	2007	2008	2009	2010	2006–2010
Organism *						
*Escherichia coli*	4/1776	4/2124	5/2202	7/2840	9/3151	29/12,093
	(0.23%)	(0.19%)	(0.23%)	(0.25%)	(0.29%)	(0.24%)
*Proteus mirabilis*	0/115	0/132	0/142	2/234	1/220	3/843
	(0%)	(0%)	(0%)	(0.85%)	(0.45%)	(0.36%)
*Klebsiella* spp.	0/484	1/561	2/562	2/807	3/823	8/3237
	(0%)	(0.18%)	(0.36%)	(0.25%)	(0.36%)	(0.25%)
*Salmonella enterica*	0/7	0/6	0/10	0/9	0/20	0/52
	(0%)	(0%)	(0%)	(0%)	(0%)	(0%)
*Shigella* spp.	0/1	0/2	0/7	0/6	0/6	0/22
	(0%)	(0%)	(0%)	(0%)	(0%)	(0%)
Totals	4/2383	5/2825	7/2923	11/3896	13/4220	40/16,247
	(0.17%)	(0.18%)	(0.24%)	(0.28%)	(0.31%)	(0.25%)

* Number of positive compared to screened isolates (%)

**Table 2:** Distribution of species and molecular types of plasmid-mediated AmpC beta-lactamase- and ESBL-producing Enterobacteriaceae (each 39 patients).

	pAmpC (n = 39)			ESBL (n = 39)		
Species	Patients, n (%)	Molecular type	Molecular type, n (%)	Patients, n (%)	Molecular type	Molecular type, n (%)
*Escherichia coli*	29 (74) *	CMY-2	26 (90)	31 (79)	CTX-M	25 (81)
		CMY-31	1 (3)		TEM, SHV	3 (10)
		ACC-1	1 (3)		n.a.	3 (10)
		DHA-1	1 (3)			
*Klebsiella pneumoniae*	6 (15) *	DHA-1	5 (83)	7 (18)	CTX-M	5 (71)
		CMY-2	1 (17)		TEM, SHV	2 (29)
*Klebsiella oxytoca*	1 (3)	DHA-1	1	0	—	—
*Proteus* spp.	3 (8)	CMY-2	3 (100)	1 (3)	TEM	1

Abbreviations: pAmpC, plasmid-mediated AmpC beta-lactamase; ESBL, Extended-Spectrum Beta-Lactamase; n.a., not available

* In one patient both *E*. *coli* and *K*. *pneumoniae* were isolated; this patient counts only once for the *E*. *coli* group

The majority of patients was female (62%) and median age was 59 years (range 21–87) (table 3). Half of the cases and controls were detected in the medical and one third in the surgical department, mainly in urology. 62% of pAmpC and 79% of ESBL patients were treated as inpatients. Co-morbidities were highly prevalent in both populations: 72% in pAmpC and 82% in ESBL carriers, respectively (p = 0.821).

**Table 3:** Characteristics of patients carrying plasmid-mediated AmpC beta-lactamase- and ESBL-producing Enterobacteriaceae (each 39 patients); univariate logistic regression analysis to define risk factors for plasmid-mediated AmpC beta-lactamase producers.

Characteristic	pAmpC (n = 39)	ESBL (n = 39)	p-value	OR (95% CI)
Demographics:				
Age, years (median)	59 (21–87)	60 (20–88)	*	*
Male, n (%)	15 (38)	15 (38)	1.000	1.00 (0.40–2.49)
Nationality, n (%):			0.994	
Swiss	23 (59)	24 (62)		0.90 (0.36–2.22)
South European	9 (23)	8 (21)		1.16 (0.40–3.41)
North European	2 (5)	2 (5)		1.00 (0.13–7.48)
Other	5 (13)	5 (13)		1.00 (0.27–3.77)
Admission from, n (%):			0.314	
Home	32 (82)	33 (85)		0.83 (0.25–2.74)
Nursing home	4 (10)	1 (3)		4.34 (0.46–40.74)
Another hospital	3 (8)	5 (13)		0.57 (0.13–2.56)
Period before detection of MRE, n (%):				
Hospitalization within ≤ 30 days	18 (46)	10 (26)	0.059	2.49 (0.96–6.46)
Antibiotic exposure within ≤ 30 days ‡	20 (63)	12 (36)	0.035	2.37 (0.94–5.98)
Antibiotic exposure at the time of MRE detection	16 (41)	15 (38)	0.817	1.11 (0.45–2.76)
Co-morbidities, n (%):				
Any	28 (72)	32 (82)	0.282	0.56 (0.19–1.63)
Cardiovascular	20 (51)	19 (49)	0.821	1.10 (0.46–2.69)
Immunosuppression §	12 (31)	6 (15)	0.107	2.44 (0.81–7.37)
Chronic renal failure (eGFR <60ml/min.)	9 (23)	12 (31)	0.472	0.67 (0.25–1.85)
Abdominal	7 (18)	6 (15)	0.761	1.20 (0.39–3.97)
Diabetes mellitus	7 (18)	7 (18)	1.000	1.00 (0.32–3.18)
Pulmonary (mainly COPD)	5 (13)	7 (18)	0.530	0.67 (0.19–2.33)
Neoplasia	3 (8)	8 (21)	0.104	0.32 (0.08–1.32)
McCabe Score, n (%) †:			0.245	
1	22 (56)	24 (62)		0.80 (0.33–2.00)
2	16 (41)	11 (28)		1.77 (0.69–4.56)
3	1 (3)	4 (10)		0.23 (0.02–2.16)
Time when MRE was detected, n (%):				
Treating department:			*	*
Internal medicine	17 (44)	19 (49)		
Surgery	12 (31)	13 (33)		
Gynaecology/obstetrics	6 (15)	4 (10)		
Intensive care unit	4 (10)	3 (8)		
Outpatient treatment	15 (38)	8 (21)	0.082	2.42 (0.88–6.65)
Devices at detection:				
No device	21 (54)	23 (59)	0.650	0.81 (0.33–1.99)
Nasogastric tube	9 (23)	0	0.002	Undefined
Urinary catheter	6 (15)	12 (31)	0.107	0.41 (0.14–1.23)
Central venous catheter	1 (3)	1 (3)	1.000	1.00 (0.06–16.57)
Multiple devices (≥2)	2 (5)	3 (8)	0.644	0.65 (0.10–4.11)
Source of MRE isolate, n (%):				
Urinary tract	27 (69)	30 (77)	0.444	0.67 (0.25–1.85)
Abdomino-rectal	6 (15)	5 (13)	0.745	1.24 (0.35–4.45)
Pulmonary	2 (5)	2 (5)	1.000	1.00 (0.13–7.48)
Bone	2 (5)	1 (3)	1.000	2.05 (0.18–23.63)
Primary sepsis	1 (3)	1 (3)	1.000	1.00 (0.06–16.57)
Superficial wound swab	1 (3)	0	1.000	Undefined
Acquisition of MRE isolates, n (%):				
Community-acquired	18 (46)	17 (44)	0.820	1.10 (0.45–2.70)
Nosocomial	13 (33)	15 (38)	0.637	0.80 (0.32–2.02)
Healthcare-associated	8 (21)	4 (10)	0.347	2.26 (0.62–8.23)
Undeterminable	0	3 (8)	0.240	Undefined
Clinical significance of MRE isolate, n (%):				
Infection	29 (74)	20 (51)	0.035	2.75 (1.06–7.15)
Urinary tract	21 (72)	16 (80)	0.797	1.68 (0.68–4.11)
Intraabdominal	3 (10)	1 (5)	0.914	3.17 (0.31–31.85)
Osteomyelitis	2 (7)	1 (5)	1.000	2.05 (0.18–23.63)
Pneumonia	1 (3)	1 (5)	1.000	1.00 (0.06–16.57)
Primary sepsis	1 (3)	1 (5)	1.000	1.00 (0.06–16.57)
Wound infection	1 (3)	0	1.000	Undefined
Colonization	10 (26)	19 (49)	0.035	0.36 (0.14–0.94)
Median duration of hospitalization, days (range)	7 (1–82)	9 (1–105)	0.337	0.73 (0.24–2.20)

Abbreviations: pAmpC, plasmid-mediated AmpC beta-lactamase; ESBL, Extended-Spectrum beta-Lactamase; OR, odds ratio; CI, confidence interval; MRE, multidrug-resistant Enterobacteriaceae; eGFR, estimated glomerular filtration rate; COPD, chronic obstructive pulmonary disease

^*^ Matching variables

‡ Patients with known exposure status to antibiotic treatment: 32 in the pAmpC and 33 in the ESBL group

§ >10 mg prednisone equivalent daily for >3 months

† McCabe Score: 1 = non-fatal disease (supposed survival >5 years for >50% of patients with the disease), 2 = ultimately fatal disease (supposed survival 1–5 years for >50% of patients with the disease), 3 = rapidly fatal disease (supposed death within 1 year for >50% of patients with the disease)

pAmpC carriers were more commonly exposed to antibiotic treatment in the 30 days prior the detection of the MRE (63%, n = 20/32 where exposure status was known) compared with 36% of ESBL controls (n = 12/33 where exposure status was known) (p = 0.035). In univariate regression analysis this factor resulted in an OR of 2.37 (95%CI 0.94–5.98). Furthermore, patients carrying pAmpC producers were more often carriers of a NGT at the time of the MRE isolation (23% (n = 9) versus none among the controls) (p = 0.002). In the 9 patients with a NGT, pAmpC producers were identified to be community-acquired in 3, nosocomial acquired in 3 and healthcare-associated in 3 patients. For the parameter ‘hospitalization within the 30 days prior the date when the MRE was isolated’ there was a trend toward a higher hospitalization rate among cases: 46% (n = 18) of cases versus 26% (n = 10) of controls (p = 0.059), resulting in an univariate OR of 2.49 (95%CI 0.96–6.46). pAmpC carriers more often had a clinically confirmed infection (74%, n = 29) compared with 51% (n = 20) of controls (p = 0.035), corresponding to an univariate OR of 2.75 (95%CI 1.06–7.15). The infections were predominately in the urinary tract (72%, n = 21). In the multivariate regression analysis, only a clinically confirmed infection remained significantly associated with pAmpC carriage with an OR of 1.44 (95%CI 1.15–2.57). No parameter in the blood tests was able to discriminate between pAmpC and ESBL carriers (data not shown).

All isolates were susceptible to carbapenems. Differences between pAmpC and ESBL producers were found only for amoxicillin/clavulanic acid and the cephalosporins (cefuroxime, ceftriaxone and cefepime) (table 4). To amoxicillin/clavulanic acid, none of the pAmpC but 31% (n = 11/35) of the ESBL producers were susceptible (p<0.001). 26% (n = 10/38) and 87% (n = 33/38) of the pAmpC producers were susceptible to ceftriaxone and cefepime, respectively. The 100% cephalosporin resistance in ESBL was in accordance with the CLSI recommendations during the study period to report ESBL always as non-susceptible to cephalosporins independent of the test result [32]. The unexpected non-susceptibility to cefepime in five pAmpC-producing isolates was further analysed and an additional beta-lactamase of the ESBL type was found. The type of the additional ESBL in these five pAmpC producers was a CTX-M type ESBL in four cases and a SHV type ESBL in one case. With regard to susceptibility patterns toward oral antibiotics, we found susceptibility rates of 50% to fluoroquinolones and co-trimoxazole and 71% to nitrofurantoin. Fosfomycin was tested only in 10 cases and 16 controls, but susceptibility was found to be 100% for both pAmpC and ESBL producers. Forty-six percent (n = 18) of pAmpC carriers did not receive effective empiric antibiotic therapy and one neutropenic patient died from a septic shock. She received inadequate empiric treatment with piperacillin-tazobactam. On the other hand, 33% (n = 13) of controls did not receive effective empiric antibiotic therapy, but none of these patients died.

**Table 4:** Antimicrobial susceptibility data of plasmid-mediated AmpC beta-lactamase- and ESBL-producing Enterobacteriaceae (each 39 patients).

Antibiotic	pAmpC (n = 39), n (%)	ESBL (n = 39), n (%)	p-value
Amoxicillin-clavulanic acid	0 / 38 (0)	11 / 35 (31)	<0.001
Piperacillin-tazobactam	20 / 37 (54)	22 / 33 (67)	0.282
Cefuroxime	1 / 38 (3)	n.a. ‡	—
Ceftriaxone	10 / 38 (26)	n.a. ‡	—
Cefepime	33 / 38 (87) *	n.a. ‡	—
Imipenem / Meropenem	38 / 38 (100)	39 / 39 (100)	1.000
Gentamicin	32 / 38 (84)	26 /39 (67)	0.074
Fluoroquinolones	14 /38 (37)	13 / 39 (33)	0.747
Co-trimoxazole	17 / 38 (45)	15 / 39 (38)	0.576
Nitrofurantoin	27 / 38 (71)	28 / 38 (74)	0.798
Fosfomycin	10 / 10 (100) §	16 / 16 (100) §	1.000

Abbreviations: pAmpC, plasmidic AmpC beta-lactamase; ESBL, Extended-Spectrum beta-Lactamase; n.a., not available; CLSI, Clinical and Laboratory Standards Institute

Percentages were calculated using susceptible isolates as nominator and all tested isolates as denominator, which was different for each antibiotic

* pAmpC producers normally are susceptible to cefepime. In five patients additional ESBL-production was documented partially explaining resistance

‡ Not available in accordance with the CLSI recommendations during the study period to report ESBL always as non-susceptible to cephalosporins

§ Susceptibility pattern of the VITEK did not include fosfomycin during the study period, therefore only a part of the isolates was tested (mostly on request by the treating physician)

## Discussion

pAmpC-producing Enterobacteriaceae are emerging pathogens [27]. Although the prevalence in this study was low, it is increasing over time. Similar increases recently reported from veterinary medicine and food industry suggest a true rise [9,33]. Furthermore, underreporting is likely, as pAmpC producers are difficult to identify, often confounded with ESBL producers and corresponding diagnostic procedures are costly and time-consuming. A clinical profile defining a high-risk population for pAmpC carriage would be desirable, such that the additional microbiological workload can be applied to a subset of patients [8]. We were unable to identify clinical and blood test-associated risk factors. Compared with ESBL controls, pAmpC carriage was more commonly associated with clinically confirmed infections and with previous antibiotic exposure. Similar to ESBL carriers, prevalence of co-morbidities was high and almost 50% of cases were community-acquired. Therefore, the identification of pAmpC producers remains a key issue for microbiology laboratories. As pAmpC producers are often associated with infections their identification is important not only for therapeutical and epidemiological reasons, but also for planning infection control measures.

Only a few case control studies have compared pAmpC and ESBL carriers [13,23,25,34]. Contrary to our study, which included in- and outpatients as well as infected and colonized patients, all of them focused on infected inpatients. Overall their findings support our conclusion, namely that patients cannot be easily distinguished by clinical or laboratory parameters [13,23,25,34]. Based on our results, pAmpC and ESBL carriers are elderly, in more than 25% of non-Swiss origin and admitted mainly from home. In both groups of MRE carriers an elevated burden of co-morbidities (more than 70%) was detected, further underscored by the finding of a McCabe score >1 in more than 40% of our patients, suggesting that pAmpC and ESBL carriers may originate from a similar source population. We found more clinically documented infections caused by pAmpC than by ESBL producers. It remains to be determined in future studies whether pAmpC producers are more virulent than ESBL producers.

Two clinically relevant factors for the acquisition of pAmpC producers were identified, in particular antibiotic exposure in the month before the pAmpC identification and indwelling NGT at the time of the pAmpC identification. This is in line with other publications, where mainly the exposure to fluoroquinolones and broad-spectrum cephalosporins were identified as risk factors for pAmpC carriage, as were UC in addition to NGT [13,17,34]. Furthermore, pancreato-biliary tract diseases and previous hospitalizations were identified as risk factors for pAmpC carriage [13,17,18,23,25,34]. In our analysis previous hospitalizations within 30 days prior to the isolation of the MRE did not reach statistical significance.

Patients carrying pAmpC producers often suffer from multiple co-morbidities leading to repetitive and prolonged hospitalizations with an increased risk for nosocomial pAmpC acquisition. However, we consider 50% of pAmpC acquisitions to be non-nosocomial, but likely community-acquired, which is similar to ESBL carriage [35]. Another recent Swiss publication is in line with our results concerning the community-acquisition of pAmpC producers. In specialized outpatient clinics a high prevalence (12.5%) of pAmpC producers was found [15]. The high prevalence in non-nosocomial settings rises multiple questions, such as the possibility of the exchange of p*ampC* genes between hospital and community (horizontal gene transfer) [24], and the source of the community-acquired pAmpC strains. Similar to ESBL producers, an animal reservoir with consecutive spread through the food chain seems likely. A screening study in healthy broiler chicken in Germany found pAmpC-producing Enterobacteriaceae in 52.9% and 56.9% of carcasses and rectal swabs, respectively [9]. Swiss studies found pAmpC-producing Enterobacteriaceae in 12.5% of pig nostrils and 25.0% of broilers [33,36]. Similar information has been published by the European Food Safety Authority: (http://www.efsa.europa.eu/en/efsajournal/pub/2322.htm). These findings suggest that measures to contain antibiotic use in livestock are strongly warranted. In addition, acquisition from field surfaces and pets has been described [37,38,39,40].

46% of our cases were community-acquired, quite different from published studies, where mostly nosocomial acquisition was described in 43%- 96% and healthcare-associated acquisition in 17%- 41% [17,18,23]. The discrepancy can be explained by differences in the study populations. Other studies mainly included infected inpatients. Furthermore, the higher consumption of broad-spectrum antibiotics in the countries where these publications originated (Spain and Korea) might select for MRE. But the present data might also show the effect of our stringent isolation policy to contain MRE, including contact isolation with a twice weekly on-site monitoring of compliance by infectious diseases practitioners.

Many of the pAmpC producers were not susceptible to multiple antibiotic classes. Susceptibility to carbapenems usually is not affected; therefore they are considered the treatment of choice. In contrary to ESBL, pAmpC producers are not susceptible to beta-lactamase inhibitors such as clavulanic acid or tazobactam [4,16]. One neutropenic patient died from septic shock. She received inadequate empiric treatment with piperacillin-tazobactam. Inappropriate empirical antibiotic therapy is not uncommon and associated with higher mortality [18,34]. Although susceptibility to cefepime is usually not affected, we do not recommend using this agent empirically for severe infections. A reduced permeability in combination with pAmpC can result in resistance to cefepime, and in up to two thirds of pAmpC producers there is a co-existing ESBL [13,41]. We detected in 13% a concomitant ESBL production, which is in line with other publications [41,42,43]. This co-existence of pAmpC and ESBL makes the phenotypic interpretation of susceptibility tests even more challenging and may account for numerous undetected pAmpC producers. During our study period cephalosporins were required to be reported as non-susceptible in ESBL producers. Nowadays, routine ESBL testing is no longer necessary for reporting results and one can report as measured according to both EUCAST and CLSI [32,44]. Non-susceptibility to fluoroquinolones and cotrimoxazole was found in 63% and 55%, respectively. Other studies found even higher resistance rates [13,18]. Because pAmpC-producing strains cause urinary tract infections in >70%, our resistance data support the strategy to favour nitrofurantoin and fosfomycin rather than fluoroquinolones and co-trimoxazole for non-invasive urinary tract infections. Because the susceptibility pattern of the VITEK did not include fosfomycin during the study period, susceptibility tests were only performed on request by the treating physician. In addition, fosfomycin is only registered in Switzerland to treat uncomplicated urinary tract infections. Susceptibility to ertapenem was not generated for this study, but increased minimal inhibitory concentrations in pAmpC-producing strains have been described [34].

We acknowledge the following limitations. The small number of clinical isolates limited the statistical power of the study, which could explain why we were unable to define a clinical profile associated with carriage of pAmpC-producing strains. But we could not find an appropriate number of matched controls. Overmatching is an inherent issue in case control studies: however when re-analyzing the dataset without the matching variable treating medical department (in particular “urology”), the results were similar. ESBL producers are common in the urology department, and therefore, we were unable to find more controls leaving out this matching criterion. The identification of pAmpC producers is challenging and missed isolates cannot be excluded, leading to underestimation of the prevalence. Currently there are no CLSI guidelines for pAmpC detection, and EUCAST only recently published an algorithm for the detection of acquired AmpC in Enterobacteriaceae [44]. Nevertheless, this algorithm was already followed by our microbiology laboratory during the study period and the only objection is that this algorithm will not detect ACC-1, a plasmid-mediated AmpC that does not hydrolyse cefoxitin. Our pAmpC detection strategy is further supported by a recently published Dutch study which analysed different screening methods for pAmpC detection and found the combination of reduced sensitivity to third generation cephalosporins and cefoxitin to generate the best specificity for phenotypic pAmpC screening [14]. Furthermore, the study of Pérez-Pérez et al. showed that AmpC multiplex PCR had discriminatory power to distinguish between the presence of known transferable *ampC* genes and suspected hyperproducing isolates of *E*. *coli* [7]. The strengths of the study include that we explicitly did not use screening data, but routine clinical specimens sent to the microbiology laboratory for analysis. By including in- and outpatients as well as infected and colonized patients, we covered a broad and representative spectrum of patients. Furthermore, we have very complete microbiological and clinical data for both groups of MRE.

In conclusion, we were unable to define a clinical profile that would allow targeted screening for pAmpC-producing Enterobacteriaceae when compared to ESBL carriers. Detection of pAmpC is difficult, time consuming and costly, and strains are commonly misclassified as ESBL. Because empiric antimicrobial therapy was inappropriate in more than 30%, rapid identification of pAmpC carriers is needed, to introduce appropriate therapy and infection control precautions. New microbiological methods are therefore required to simplify rapid and reliable detection of pAmpC carriers.
